# Diagnostic yield or diagnostic accuracy estimation?

**DOI:** 10.1183/13993003.02778-2025

**Published:** 2026-04-16

**Authors:** Oren Fruchter

**Affiliations:** The Pulmonary Division, Edith Wolfson Medical Center and Gray Faculty of Medical and Health Sciences, Tel Aviv University, Tel Aviv, Israel

## Abstract

I read with great interest the recent publication by Deng *et al*. [1] titled “Ultrasonic elastography-guided pleural biopsy *versus* traditional thoracic ultrasound-guided pleural biopsy for the diagnosis of pleural effusion: a multicentre, randomised trial.”

## Shareable abstract

**Diagnostic accuracy estimation is not a substitute for true diagnostic yield** https://bit.ly/4t7V1z7

*To the Editor:*

I read with great interest the recent publication by Deng *et al*. [1] titled “Ultrasonic elastography-guided pleural biopsy *versus* traditional thoracic ultrasound-guided pleural biopsy for the diagnosis of pleural effusion: a multicentre, randomised trial.”

The authors reported that the “diagnostic yield” of ultrasonic elastography-guided pleural biopsy was significantly higher than traditional thoracic ultrasound-guided pleural biopsy: 87.83% *versus* 76.99% (p=0.032).

The “diagnostic yield” was calculated as the proportion of patients who obtained a subsequent definitive diagnosis (benign or malignant) calculated as: (true positives + true negatives)/total cases. To note, the authors state that the follow-up period to establish true benignity in several cases was at least 12 months.

Ost *et al*. [2] suggested a methodological framework that lays a foundation for a clear distinction between diagnostic yield and diagnostic accuracy when evaluating a novel diagnostic test in pulmonary medicine. Other authors have used that methodology for evaluating results of diagnostic bronchoscopy [3, 4].

Diagnostic accuracy refers to results deemed diagnostic (positive or negative) that are confirmed to be accurate after a follow-up period.

Diagnostic yield should refer only to the proportion of cases in which the index biopsy provides a pathological result allowing for confident management of the patient without relying on confirmatory follow-up clinical data and/or subsequent additional biopsies.

Deng *et al*. [1] essentially report diagnostic accuracy estimates rather than true diagnostic yield since the index biopsy was deemed as diagnostic or nondiagnostic based on follow-up clinical data not available at the time of the initial procedure.

A true diagnostic yield would entail only data obtained from the initial biopsy. Hence in the current study the numerator (true positives + true negatives) should include only cases in which the index pleural biopsy provided conclusive specific benign diagnosis, such as sarcoidosis or tuberculosis, or definitive malignant diagnosis, such as metastatic adenocarcinoma or mesothelioma.

While reanalysis of data and establishing the true diagnostic yield of elastography-guided pleural biopsy obtained in the current study is not possible, I believe further studies using the current report as a benchmark for comparison should incorporate these points into consideration.
